# Low Back Pain Reduces the Amount of Lumbopelvic Movement During Lower Limb Motion: A Cross-Sectional Study of Soccer Players

**DOI:** 10.7759/cureus.86694

**Published:** 2025-06-24

**Authors:** Toshimitsu Ohmine, Atsuki Kanayama, Saki Yamamoto, Keita Sasada, Kazuma Senzaki, Yoshihiko Kawamoto, Koji Nonaka, Akira Iwata

**Affiliations:** 1 Department of Physical Therapy, Faculty of Comprehensive Rehabilitation, Osaka Prefecture University, Habikino, JPN; 2 Department of Rehabilitation Sciences, Division of Physical Therapy, Kansai University of Welfare Sciences, Kashiwara, JPN; 3 Graduate School of Rehabilitation Science, Osaka Metropolitan University, Habikino, JPN; 4 Department of Rehabilitation, Faculty of Health Sciences, Naragakuen University, Nara, JPN

**Keywords:** inertial measurement unit, low back pain, lower limb motion task, lumbopelvic movement, soccer players

## Abstract

Introduction: Low back pain (LBP) is common among soccer players and may influence lumbopelvic movement strategies. This study aimed to examine the effect of LBP on the amount of lumbopelvic movement during lower limb motion tasks performed at both high and low speeds.

Methods: Ninety-nine high school soccer players from Osaka Metropolitan University, Osaka, Japan, participated in this study. They performed a task in which they raised the dominant leg by flexing the hip and knee joints from a standing position until the hip reached approximately 90 degrees of flexion, under both high (400°/s) and low (100°/s) speed conditions. An inertial measurement unit (IMU) attached to the lumbar spine measured total lumbopelvic movement and movement in three directions (rotation, flexion-extension, and side bending) based on the angular velocity. Participants were classified into the LBP and non-LBP groups based on the self-reported presence of the current LBP. Group differences were analyzed using independent t-tests and Mann-Whitney U tests.

Results: Under low-speed conditions, the LBP group exhibited reduced total lumbopelvic movement (p < 0.02; d = 0.56), particularly for rotation (p < 0.01; d = 0.73). No significant differences were observed under high-speed conditions.

Conclusion: Soccer players with LBP demonstrate reduced lumbopelvic movement, particularly in rotation, under low-speed conditions. This may reflect a motor control strategy to avoid pain by limiting lumbopelvic movement. However, no significant differences were observed under high-speed conditions, possibly because high-velocity tasks require greater lumbopelvic movement, making it difficult to restrict movement even in the presence of pain. Rapid lower limb motions, such as kicking or sprinting, may involve greater lumbopelvic movement. Consequently, training and rehabilitation programs for soccer players with LBP should consider the potential for symptom aggravation under high-speed conditions.

## Introduction

Low back pain (LBP) is a common issue among soccer players, with elite-level athletes reporting annual prevalence rates of 31.4-57% [1,2]. Moreover, LBP often forces athletes to cease sports activities [3]. In soccer, actions such as sprinting [4] and ball kicking [5] are frequently linked to LBP due to the extensive lumbopelvic movement required. Sairyo et al. demonstrated, through finite element modeling, that excessive lumbar spine and pelvic extension with rotational motion increases shear and rotational stress on the intervertebral joints [6]. Therefore, while the importance of core function in providing stability and generating force is well recognized, controlling excessive lumbopelvic movement during lower limb motions is considered particularly important for preventing LBP [7].

The relationship between lumbopelvic movement and LBP during lower limb motions has previously been studied using various methods. For example, static quantitative tests include the Active Straight Leg Raising test, which evaluates the control of lumbar and pelvic rotation in the transverse plane during a straight-knee leg raise [8], and the prone hip extension test, which assesses control of lumbar and pelvic rotation during a single-leg raise from a prone position [9]. However, studies using the Active Straight Leg Raising [10] or prone hip extension test [11] have found no significant differences in lumbar or pelvic displacement between individuals with LBP and healthy athletes.

In contrast to static tests performed in a recumbent position, dynamic qualitative tests, such as the standing knee lift test, evaluate lumbopelvic movement while standing [12]. This test evaluates the presence or absence of some specific lumbopelvic movements when one leg is slowly raised. However, a study examining the potential causal relationship between standing knee lift test scores and the occurrence of LBP in athletes found no significant correlation between the two [12].

In sports, excessive lumbar spine and pelvic movements are believed to contribute to LBP [5]. However, previous studies involving athletes have not clearly demonstrated an association between the magnitude of lumbopelvic movement during lower limb tasks and LBP, whether assessed through static or dynamic tests. The absence of a clear association may be due to the low-speed tasks or the use of qualitative assessment methods [10,12]. Therefore, to clarify the relationship between LBP and the amount of lumbopelvic movement in athletes, it is necessary to (1) assess lumbopelvic kinematics during high-speed lower limb movements and (2) quantitatively assess the amount of lumbopelvic movement.

This study used inertial measurement units (IMUs) to quantitatively measure the amount of lumbopelvic movements in soccer players performing lower limb tasks at high and low speeds. The primary objective was to determine how LBP affects lumbopelvic movement in these conditions. Increased lumbopelvic movement may elevate stress on the lumbar tissue (e.g., ligaments and intervertebral discs) and contribute to LBP [13,14]. Thus, we hypothesized that soccer players with LBP would exhibit greater lumbopelvic movement than those without LBP during high-speed conditions, whereas no significant differences would be observed during low-speed conditions. These findings could help develop targeted screening and intervention strategies to prevent and manage LBP in soccer players, contributing to both LBP prevention and performance enhancement.

## Materials and methods

Study design

This cross-sectional study investigated lumbopelvic movement during lower limb motion tasks in high school soccer players with and without LBP.

Participants

Participants included 99 male high school soccer players, aged 16 to 17 years, from Osaka Metropolitan University, Osaka, Japan. The study targeted three public high school teams affiliated with a local soccer association. Coaches were contacted to arrange study explanations, after which players voluntarily enrolled. Inclusion criteria were (1) a hip flexion angle of at least 100° in both legs, (2) the ability to participate in regular practices and games, (3) having received regular at least annual medical checkups with no serious disorders identified by their physicians, and (4) having undergone mental health screening with no concerns identified by the psychologist.

This study was conducted in accordance with the ethical principles outlined in the Declaration of Helsinki and approved by the Human Ethics Committee of Osaka Prefecture University (approval number 2022-131). Informed consent was obtained in writing and verbally from all participants for study participation and publication.

Demographic data and LBP score

Participants provided self-reported demographic data, including age, number of years of competitive experience, playing position, and leg dominance. Leg dominance was defined as the preferred kicking leg [15]. Height and weight were measured using a standard stadiometer and scale. Current LBP severity was assessed using the Micheli Functional Scale [16] and the Roland-Morris Disability Questionnaire [17]. LBP history over the past three years was recorded. Current LBP was defined as pain lasting >1 week or causing cessation of soccer activities for >3 days.

Measurement of the amount of lumbopelvic movement

Measurements and analyses were performed using only the dominant leg.

Experimental Setup

**Figure 1:**
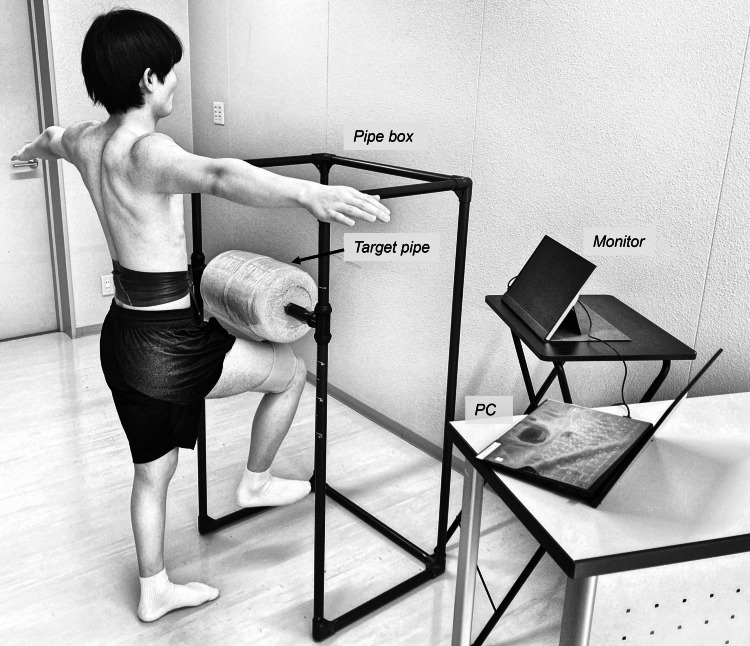
Equipment and measurement environment Abbreviation: PC, personal computer. This figure is an original creation by the authors.

A pipe box measuring 60 cm × 45 cm × 200 cm was constructed from plastic pipes and used to limit and standardize the range of leg motion of participants (Figure 1). Participants stood in front of this pipe box and performed lower limb motion toward a height-adjustable target pipe wrapped in a soft cushion, serving as a reference point for motion consistency. Real-time visual feedback from a monitor enabled precise velocity adjustments.

Measurement Preparation

**Figure 2:**
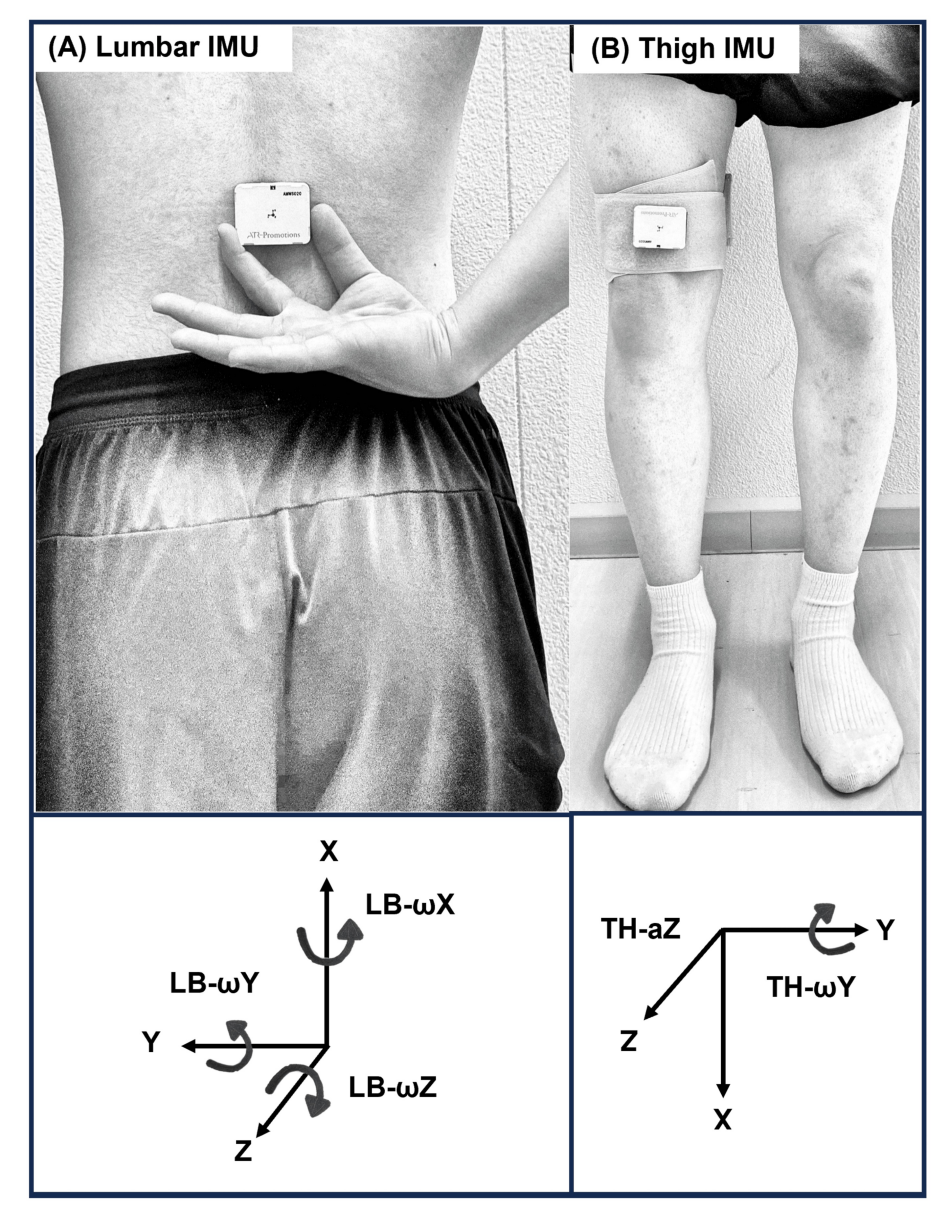
Placement and orientation of the two inertial measurement units (A) Lumbar IMU: Affixed above the third lumbar spinous process. The IMU’s axes are oriented as follows: X (upward), Y (lateral), and Z (backward). LB-ωX (angular velocity around the X-axis) indicates lumbopelvic rotation, LB-ωY (around the Y-axis) indicates flexion–extension, and LB-ωZ (around the Z-axis) indicates side flexion. (B) Thigh IMU: Affixed 10 cm proximal to the patella. The IMU’s axes are oriented as follows: X (downward), Y (lateral), and Z (forward). TH-ωY (angular velocity around the Y-axis) measures hip flexion velocity, and TH-aZ (acceleration along the Z-axis) detects thigh contact. Abbreviations: a, acceleration; IMU, inertial measurement unit; ω, angular velocity; LB, lumbar; TH, thigh. This figure is an original creation by the authors.

First, the height of the target pipe was individually adjusted so that each participant’s thigh was parallel to the floor when raising the leg. Second, two IMUs (AMWS020; ATR-Promotions Inc., Kyoto, Japan; ±16 g; sampling rate, 500 Hz) were utilized; each integrated a triaxial accelerometer and triaxial gyroscope sensor. Figure 2 illustrates the orientations of both IMUs. One IMU was mounted at the height of the third lumbar spinous process to measure lumbopelvic movement near the participant’s center of mass (lumbar IMU; Figure 2A) [18,19]. It was securely affixed to the skin with cotton tape, with Theraband (Theraband, Akron, Ohio, USA) wrapped over it, around the participant’s waist. The other IMU (thigh IMU) was affixed to the motion leg, positioned 10 cm proximal to the patella with a Velcro strap (Figure 2B). IMU data were wirelessly transmitted via Bluetooth® (Bluetooth SIG, Inc., Kirkland, Washington, USA) to a personal computer (MS-14D2; Micro-Star International Co., Ltd, New Taipei City, Taiwan), displaying the Y-axis angular velocity waveform of the thigh IMU on a monitor. Both IMUs were synchronized before measurement.

Lower Limb Motion Task

The participants stood in front of the pipe box with both shoulders abducted to 90°, allowing clear observation of the lumbopelvic region. They lifted the dominant leg toward the target pipe cushion by flexing the hip to approximately 90 degrees and bending the knee (Figure 1). The participants were instructed to (1) avoid initiating the leg lift using recoil motions, (2) maintain motion speed without slowing down until contacting the cushion, and (3) maintain a single-leg stance for three seconds after contact.

Lower limb motion velocity was quantified by measuring the thigh’s Y-axis angular velocity (hip flexion velocity) using the thigh IMU (Figure 2B). The participants adjusted leg velocity based on real-time feedback, displayed as an angular velocity waveform on the monitor, and targeting either low-speed (approximately 100°/s) or high-speed (approximately 400°/s) conditions. The order of speed conditions was randomized.

The participants completed five practice trials per condition. The task was repeated until three successful trials per condition were recorded. A trial was deemed unsuccessful if the participant (1) initiated the leg lift with recoil, confirmed visually or through angular velocity waveforms, or (2) failed to maintain a three-second single-leg stance after making contact with the cushion.

Analysis of Lumbopelvic Movement

**Figure 3:**
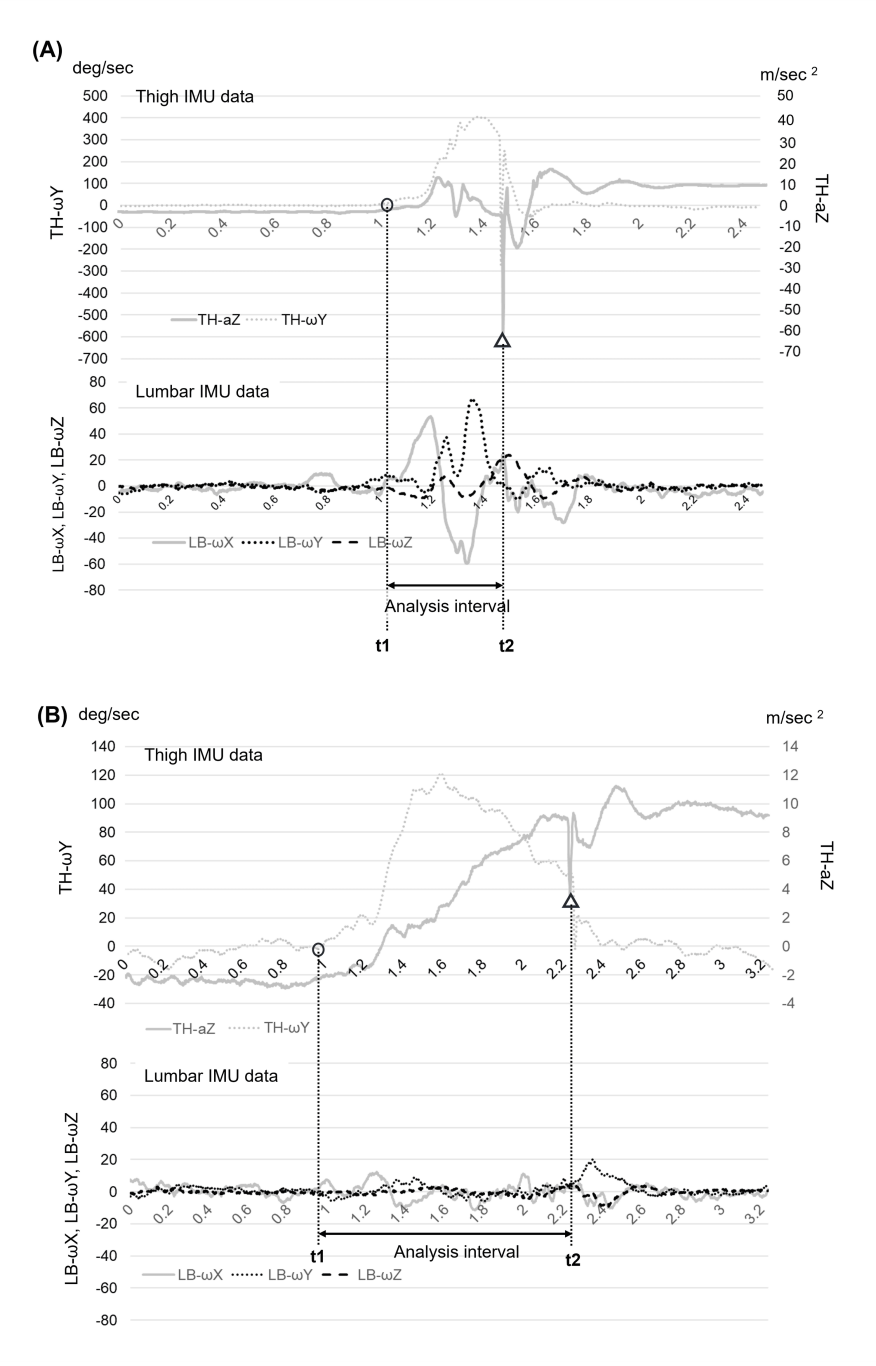
Typical waveforms and determining the analysis interval Both graphs display representative thigh (upper panels) and lumbar (lower panels) IMU data under high- (A) and low-speed (B) conditions. The start time (indicated by a circle) represents when TH-ωY exceeds 5°/s, marking the initiation of lower limb motion. The end time (indicated by a triangle) represents when TH-aZ shows a sharp negative peak, indicating thigh contact and motion termination. The interval between the two dotted lines represents the analysis period. Abbreviations: a, acceleration; IMU, inertial measurement unit; LB, lumbar; ω, angular velocity; TH, thigh.

Figure 3 presents representative waveforms from the lumbar and thigh IMUs. The analysis interval was defined using thigh motor data from the thigh IMU. The start of the interval (t1) was defined as the time when the angular velocity around the Y-axis (representing hip flexion) of the thigh IMU first exceeded 5°/s. The end of the interval (t2) was defined as the time when the Z-axis acceleration (TH-aZ) of the thigh IMU exhibited a clear negative peak, which reflected a sudden deceleration upon thigh contact with the cushion. If the angular velocity of the thigh IMU exceeded −5°/s (negative direction) before initiating the lower limb motion, the attempt was considered invalid due to recoil motion and was excluded from the analysis.

The amount of lumbopelvic movement was calculated based on angular velocities measured around the three axes of the lumbar IMU: LB-ωX, LB-ωY, and LB-ωZ, which corresponded approximately to lumbopelvic rotation, flexion-extension, and side flexion movements, respectively [20,21]. For each attempt, the amount of movement in each direction was separately calculated by integrating the absolute values of these angular velocities over the analysis interval (t1 to t2) as follows:

Rotation movement (LB rot):

$LB rot=\int_{t1}^{t2}|f(LB-\omega\text{X})|dt$ (1)

Flexion-extension movement (LB flex/ext):

$LB flex/ext =\int_{t1}^{t2}|f(LB-\omega\text{Y})|dt$ (2)

Side flexion movement (LB side flex):

$LBside flex =\int_{t1}^{t2}|f(LB-\omega\text{Z})|dt$ (3)

Total lumbopelvic movement was calculated by integrating the vector magnitudes of angular velocities over the analysis interval (t1 to t2), as shown in Equation (4):

$Total =\int_{t1}^{t2}\sqrt{(LB-\omega\text{X})^2+(LB-\omega\text{Y})^2+(LB-\omega\text{Z})^2}dt$ (4)

All participants completed at least two valid trials under both speed conditions. The final lumbopelvic movement values were obtained by averaging the values of two trials. If all three attempts were valid, the first two were used to ensure consistency across participants.

Reliability and minimal detectable change

Test-retest reliability was evaluated using the intraclass correlation coefficient (ICC (1,2)) based on data obtained from a separate sample of 15 participants. ICC values for all variables indicated good to excellent reliability. The minimal detectable change (MDC) was calculated using the standard error of measurement derived from the same dataset. Detailed results are shown in Table 1.

**Table 1:** Intraclass correlation coefficient (ICC) and minimal detectable change (MDC) values for each variable Abbreviations: flex/ext, flexion–extension movement; LB, lumbopelvic; rot, rotation movement; side flex, side flexion movement.

	ICC (1,2)	MDC
High-speed condition		
LB rot	0.95	0.47
LB flex/ext	0.85	1.16
LB side flex	0.60	0.72
Total	0.94	0.58
Low-speed condition		
LB rot	0.89	1.07
LB flex/ext	0.77	1.82
LB side flex	0.88	0.65
Total	0.91	1.16

Sample size

The sample size for an independent samples t-test was calculated using G*Power 3.1 (Heinrich Heine University, Düsseldorf, Germany) with a statistical power of 0.80, an alpha level of 0.05, and an effect size of 0.80. Based on previous research [1,22], we assumed an LBP prevalence of 30%. The required sample size was 19 players in the LBP group and 43 in the non-LBP group, totaling 62 players. Accounting for a potential 20% data loss, we determined that at least 78 participants should be enrolled.

Statistical analysis

The participants were divided into the LBP and non-LBP groups based on the presence or absence of current LBP. Normality of all quantitative variables was assessed using the Shapiro-Wilk test. Independent samples t-tests were used for normally distributed variables, and Mann-Whitney U tests were applied to non-normally distributed variables. Chi-square tests or Fisher’s exact tests were performed to analyze qualitative (categorical) variables, depending on the expected cell counts. Statistical significance was set at p < 0.05. Effect sizes (Cohen’s d and r values) were also calculated. All statistical analyses were conducted using IBM SPSS Statistics for Windows, version 28.0 (released 2021, IBM Corp., Armonk, NY).

Use of a large language model in the writing process

During manuscript preparation, the authors used ChatGPT (GPT-4o, OpenAI, San Francisco, California) to refine language and improve clarity. After using GPT-4o, the authors reviewed and edited the content as needed and take full responsibility for the publication's final version.

## Results

**Figure 4:**
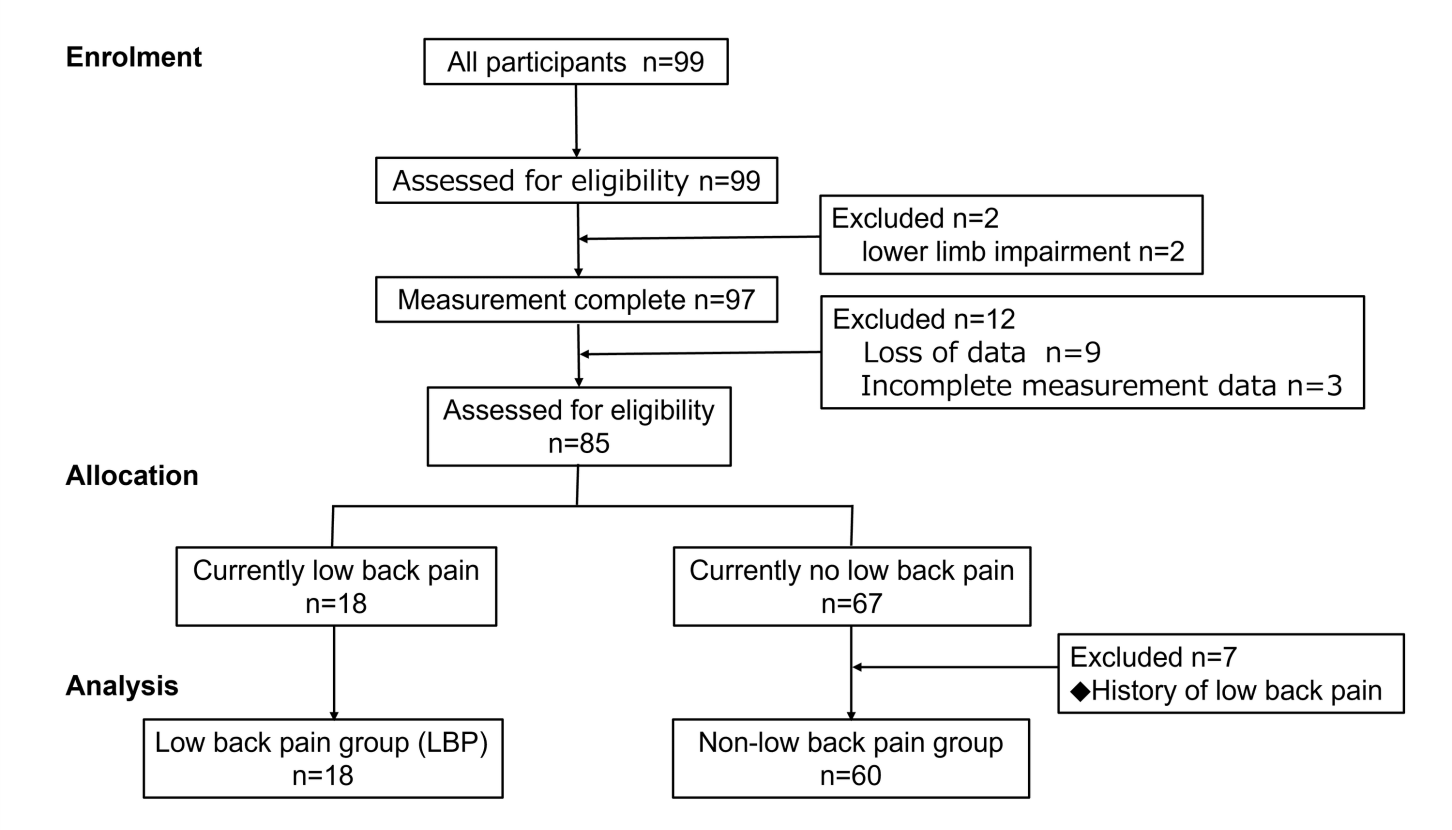
Flow diagram showing participant enrollment and classification

Of the 99 participants initially recruited, 78 (78.8%) were included in the final analysis. Among them, 18 (23.1%) were classified into the LBP group and 60 (76.9%) into the non-LBP group (Figure 4).

Table 2 presents the participant demographics and LBP scores. No significant differences between groups in age, height, weight, competitive experience, or leg dominance were observed. Micheli Functional Scale and Roland-Morris Disability Questionnaire scores indicated mild functional impairment in the LBP group.

**Table 2:** Participant demographic characteristics and low back pain scores * Mean ± standard deviation, † Median (interquartile range), ‡ Number of data, a: Independent samples t-test, b: Chi-square tests, c: Fisher’s exact test was used; no test statistic is reported. Statistical significance was determined at p < 0.05. Abbreviations: FW, forward; MF, midfielder; DF, defender; GK, goalkeeper; MFS, Micheli Functional Scale; RDQ, Roland–Morris Disability Questionnaire.

	Non-LBP (n = 60)	LBP (n = 18)	Test statistic	p-value
Age (year)*	16.2 ± 0.7	16.3 ± 0.6	t = 0.31	0.76^a^
Height (cm)^*^	171.1 ± 4.8	172.5 ± 6.6	t = 0.94	0.35^a^
Weight (kg)^*^	60.2 ± 5.6	61.3 ± 4.4	t = 0.70	0.49^a^
Years of experience (year)^*^	7.8 ± 3.5	7.9 ± 3.9	t = 0.05	0.96^a^
Position (FW/MF/DF/GK)^‡^	8 / 22 / 23 / 7	3 / 5 / 8 / 2	χ² = 0.89	0.76^b^
Dominant leg (right / left)^‡^	51 / 9	15 / 3	n/a	1.00^c^
MFS^†^ (min–max)		16 (4–48)		
RDQ^†^ (min–max)		0.5 (0–7)		

Table 3 displays lumbopelvic movement measurements during lower limb tasks under high- and low-speed conditions. In the high-speed condition, lower limb motion velocity was 409.8 ± 43.2°/s, whereas in the low-speed condition, it was 111.8 ± 21.0°/s. The LBP group exhibited significantly lower values than the non-LBP group for rotation movement (p < 0.01; d = 0.73) and total lumbopelvic movement (p = 0.02; d = 0.56) during the low-speed condition.

**Table 3:** Amount of lumbopelvic movement during lower limb tasks * Mean ± standard deviation, † Median (interquartile range), a: Mann-Whitney U test, b: Independent samples t-test, d: Cohen's d, r: Mann-Whitney's r. Statistical significance was determined at p < 0.05. Abbreviations: flex/ext, flexion–extension movement; LB, lumbopelvic; rot, rotation movement; side flex, side flexion movement.

	Non-LBP (n = 60)	LBP (n = 18)	Test statistic	p-value	effect size
High-speed					
LB rot (°)^†^	10.28 (7.66–14.50)	10.64 (7.89–13.67)	U = 526.0	0.92^a^	0.01^r^
LB flex/ext (°)^†^	6.51 (4.59–10.04)	4.53 (3.65–9.16)	U = 421.5	0.13^a^	0.17^r^
LB side flex (°)^†^	3.11 (2.63–4.06)	3.59 (2.61–5.04)	U = 482.0	0.53^a^	0.07^r^
Total (°)^*^	15.62 ± 4.66	14.63 ± 3.91	t = 0.88	0.38^b^	0.22^d^
Low-speed					
LB rot (°)^*^	8.23 ± 2.02	6.78 ± 1.78	t = 3.33	<0.01^b^	0.73^d^
LB flex/ext (°)^†^	5.03 (3.78-6.49)	4.50 (3.82–5.05)	U = 424.0	0.12^a^	0.18^r^
LB side flex (°)^†^	3.77 (2.84-4.59)	3.79 (2.65–4.92)	U = 506.5	0.64^a^	0.05^r^
Total (°)^*^	11.97 ± 2.71	10.53 ± 2.04	t = 2.34	0.02^b^	0.56^d^

## Discussion

This study examined differences in the amount of lumbopelvic movement between soccer players with and without LBP during single-leg lifting tasks at high and low velocities. Our primary finding was that soccer players with LBP exhibited significantly less lumbopelvic movement than those without LBP under low-speed conditions, whereas no significant differences were observed under high-speed conditions. Furthermore, directional analysis revealed that this reduction was specific to rotation, with no significant differences in flexion-extension or side flexion movements.

The finding of reduced lumbopelvic movement under low-speed conditions aligns with those of previous studies, which have reported reduced movement in individuals with LBP during trunk forward bending [23] and standing reach tasks [24]. These behaviors likely serve as a protective mechanism to avoid or reduce LBP. Furthermore, patients with LBP may limit movement between lumbar and sacral vertebral segments through co-contraction of trunk muscles, reducing mechanical stress [25]. Thus, participants of the LBP group in the present study may have restricted their lumbopelvic movement as a strategy to minimize discomfort.

The observed reduction in overall lumbopelvic movement in the LBP group primarily resulted from selective restriction of rotational movement. Why rotation was preferentially suppressed over flexion-extension or side flexion is worth considering. Selective restriction of rotational movement may serve as a protective strategy: spinal rotation has been strongly linked to LBP and increases the risk of occupationally related low back disorders compared to other directions of movement [26]. This may be due to the greater mechanical stress placed on facet joints and intervertebral discs during rotational movements [27]. Consequently, athletes experiencing LBP may preferentially limit rotational movements to reduce pain and prevent further tissue stress or injury. Rotational movement has been shown to compromise spinal stability more than movements in other planes; therefore, controlling rotational movements may also enhance trunk stability [28]. Thus, restricting rotational movement could represent an adaptive mechanism to minimize pain while improving trunk stability.

Our initial hypothesis assumed that increased lumbopelvic movement would lead to greater loading on the lumbar tissues, contributing to LBP [13,14]. Based on this assumption, we expected soccer players with LBP to exhibit greater lumbopelvic movement, particularly under high-speed conditions in which mechanical load would be more pronounced. However, our results contradict this hypothesis, suggesting instead that players with LBP actively restrict lumbopelvic movement to minimize pain. This tendency was particularly evident under low-speed conditions, in which control of movement was easier. These findings align with a study that reported that individuals with LBP adopted compensatory movement strategies to avoid discomfort [24].

Under high-speed conditions, no significant differences in the amount of lumbopelvic movement were observed between the LBP and non-LBP groups. While individuals with LBP may adopt pain-avoidance strategies during slow movements, motor control often deteriorates during fast movements, possibly due to impaired proprioception and reduced trunk muscle variability [29]. Therefore, under the high-speed conditions of this study, individuals with LBP appeared unable to adopt pain-avoidance strategies. Consequently, they may have increased their exercise volume, resulting in no significant difference relative to the healthy group.

This study had five limitations. First, we did not analyze specific directions of lumbopelvic movement (e.g., left rotation, right side flexion, or flexion). Because our primary aim was to examine overall lumbopelvic movement, detailed directional analysis was considered beyond the scope of this study. Second, our participants were high school soccer players aged 16-17 years. Therefore, the generalizability of these findings to other age groups, such as elementary, middle school, or university soccer players, may be limited. Nonetheless, high school players (U17) have been reported to have a higher risk of LBP compared to middle school (U15) players (odds ratio 1.66) [30]. Thus, focusing on this age group was justified. Third, although the participants in this study underwent annual psychological screenings with no abnormalities detected, psychological factors are known to have a significant influence on the onset of low back pain. As this study did not thoroughly assess these factors, it is important to acknowledge the possibility that they may have influenced the results. Fourth, although the movement tasks and velocities used in this study are closer to actual sports movements than those in previous studies, they may not fully replicate the intensity of real-world sport-specific activities. Therefore, caution is warranted when interpreting the applicability of the findings to competitive sports settings. Fifth, this study did not account for detailed joint movements (e.g., sacroiliac and intervertebral joints) or muscle activity associated with the tasks. Therefore, we cannot provide a definitive explanation for the observed reduction in lumbopelvic movement during low-speed lower limb movement tasks in soccer players with LBP.

## Conclusions

This study quantitatively evaluated the amount of lumbopelvic movement during lower limb motion performed at different velocities in soccer players with and without LBP.

Our findings demonstrate that soccer players with LBP exhibit significantly reduced lumbopelvic movement, particularly in rotation, under low-speed conditions. These results provide valuable insights that could inform targeted interventions aimed at controlling rotational movements of the lumbopelvic region, contributing to the prevention and management of recurrent LBP in soccer players. Future studies should evaluate the effectiveness of interventions designed to improve lumbopelvic movement control and examine their impact on both pain and sports performance in this population.
